# Immune Privilege of Heart Valves

**DOI:** 10.3389/fimmu.2021.731361

**Published:** 2021-08-10

**Authors:** Morgan Ashley Hill, Jennie H. Kwon, Brielle Gerry, William A. Hardy, Olivia Agata Walkowiak, Minoo N. Kavarana, Satish N. Nadig, T. Konrad Rajab

**Affiliations:** ^1^College of Medicine, Medical University of South Carolina, Charleston, SC, United States; ^2^Division of Cardiothoracic Surgery, Medical University of South Carolina, Charleston, SC, United States; ^3^Division of Transplant Surgery, Medical University of South Carolina, Charleston, SC, United States

**Keywords:** immune privilege, heart valve, heart valve allograft, transplantation, transplantation (heart)

## Abstract

Immune privilege is an evolutionary adaptation that protects vital tissues with limited regenerative capacity from collateral damage by the immune response. Classical examples include the anterior chamber of the eye and the brain. More recently, the placenta, testes and articular cartilage were found to have similar immune privilege. What all of these tissues have in common is their vital function for evolutionary fitness and a limited regenerative capacity. Immune privilege is clinically relevant, because corneal transplantation and meniscal transplantation do not require immunosuppression. The heart valves also serve a vital function and have limited regenerative capacity after damage. Moreover, experimental and clinical evidence from heart valve transplantation suggests that the heart valves are spared from alloimmune injury. Here we review this evidence and propose the concept of heart valves as immune privileged sites. This concept has important clinical implications for heart valve transplantation.

## Introduction

**Figure 1:**
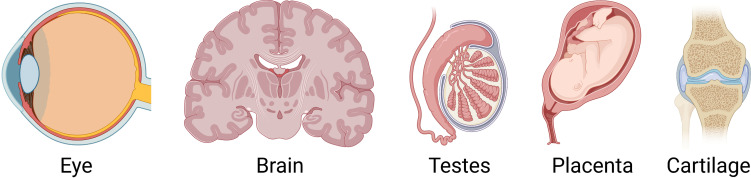
The classical immune privileged sites are the eye, central nervous system, placenta, testes and articular cartilage.

The term immune privilege was coined by Sir Peter Medawar in the 1940s to describe sites of the body in which the introduction of foreign antigens does not elicit an inflammatory immune response. While most tissues can tolerate complex inflammatory responses and immune reactions to clear infections, some tissues are unique in the fact that even minor periods of inflammation can have lasting consequences for the evolutionary fitness of the organism (1). Therefore, such vital tissues with limited regenerative capacity need to be protected from collateral damage caused by the immune response. The classical immune privileged sites are the eye, central nervous system, placenta, testes and articular cartilage (**Figure 1**). Here we provide an overview of the immune privilege of these sites, and evaluate the evidence that heart valves also have immune privilege.

## Ocular Immune Privilege

Medawar’s initial investigations of transplant immunity demonstrated that both the anterior chamber of the eye and the brain exhibit immune privilege. In his experiment, Medawar sensitized recipient rabbits to skin allografts of donor rabbits by grafting the skin of the donor onto the skin of the recipient (2). After the allografts were rejected by the recipients, Medawar used the same donor-recipient pairs and implanted skin allografts from the donor rabbits into one of three different anatomical sites on the recipients: the skin, the anterior chamber of the eye, and the left cerebral hemisphere. The allografts implanted in skin were rejected rapidly, but the allografts implanted in the anterior chamber of the eye and the left cerebral hemisphere demonstrated a reduced rate of rejection, i.e. immune privilege.

The tissues of the eye have a limited regenerative capacity, so it is thought that the function of immune privilege in the eye is an evolutionary strategy for protecting these tissues from a systemic immune response (3). The immune privilege of the eye is maintained *via* several mechanisms: the physical sequestration of alloantigen, the presence of a local anti-inflammatory milieu, and the induction of systemic immune tolerance (4). The physical sequestration of antigens is achieved through a relative absence of efferent lymphatics in the eye and the presence of the blood-retina barrier. The blood-retina barrier is composed of an outer layer of retinal pigment epithelial cells and an inner layer composed of retinal microvascular endothelial cells, both of which are upheld by tight junctions between cells (5). These layers regulate the passage of solutes into the microenvironment of the eye (5). The anti-inflammatory local microenvironment of the eye is characterized by the FasL-mediated induction of apoptosis of T cells entering the eye (6) and the production of immunomodulatory molecules such as α-MSH, TGFβ2, and neuropeptide Y (4). There is also evidence for the protective role of the inducible costimulatory molecule ICOS in corneal transplantation and maintenance of immune privilege (7). Lastly, immune privilege in the eye is mediated by the induction of systemic immune tolerance, known as anterior chamber-associated immune deviation (ACAID) (4). This tolerance against alloantigens in the eye is caused by the upregulation of regulatory T cells *via* a pathway involving the presentation of the alloantigen on F4/80+ macrophages in the spleen (4). Conversely, pathological conversion of regulatory T cells contributes to the loss of corneal immune privilege (8).

## Central Nervous System Privilege

As Medawar observed, immune privilege is also seen in the central nervous system (CNS). Similar to the eye, the tissues of the CNS are essential for survival and possess a limited regenerative capacity, thus immune privilege in the CNS prevents damage from occurring during an immune response (3). The immune privilege of the CNS is maintained through physical barriers as well as cellular and molecular mechanisms restricting traversal across these barriers (9). The physical barriers of the CNS that maintain immune privilege are the blood-brain barrier (BBB), which is composed of tight junctions between vascular endothelial cells; the blood-cerebrospinal fluid barrier (BCSFB), which is upheld by tight junctions between epithelial cells of the choroid plexus; and the glia limitans, which is composed of astrocyte foot processes and the parenchymal basement membrane on the outermost layer of the CNS (9, 10). These barriers allow antigens in the CNS to be relatively sequestered from the immune system. The CNS parenchyma generally does not contain adaptive immune cells, but the BCSFB allows low numbers of adaptive immune cells to migrate into the cerebrospinal fluid (11). Furthermore, the pericytes of the BBB regulate transmigration of immune cells across the BBB and promote an immunosuppressive environment by producing TGFβ (11, 12).

## Placental Immune Privilege

Immune privilege is also observed in the placenta during pregnancy. The placenta contains fetal antigens, which appear foreign to the maternal immune system. Immune privilege in the placenta prevents the maternal immune system from rejecting the placenta and causing spontaneous abortion. Placental immune privilege is maintained by a physical barrier that regulates the passage of maternal leukocytes, altered expression of human leukocyte antigen (HLA), and molecular mechanisms that locally inhibit T cells (13, 14). The physical barrier of the placenta is known as the maternal-fetal interface and is composed of a layer of fetal syncytiotrophoblast and maternal decidua (14). This barrier separates the fetal and maternal circulatory systems, although the fetal syncytiotrophoblast does come into contact with maternal blood. The syncytiotrophoblast is able to avoid recognition by the maternal immune system by not expressing any HLA (13). Extravillous trophoblast within the maternal decidua expresses HLA-G, which is a non-classical MHC class I antigen (15). HLA-G attenuates the immune response at the placental barrier by inhibiting maternal natural killer and T cells (16). The placental trophoblasts also have several other molecular mechanisms for altering T cell response: the expression of costimulatory B7, which promotes Treg activation; the production of indoleamine 2,3-dioxygenase (IDO), which starves T cells of tryptophan; and the expression of FasL and TNF-related apoptosis-inducing ligand (TRAIL), which induces the apoptosis of T cells (17).

## Testicular Immune Privilege

The testis is another anatomical site possessing the properties of immune privilege. Spermatogenesis within the testis produces highly immunogenic spermatozoa, which express different antigens than somatic cells. Testicular immune privilege serves to prevent the systemic immune response from impacting fertility. Immune privilege in the testis occurs because of its unique physical structure, an anti-inflammatory local environment, and systemic immune tolerance (13). Antigens within the innermost portion of the seminiferous tubules of the testis are physically sequestered from the immune system by the blood-testis barrier, which is formed by adjacent Sertoli cells tethered *via* specialized junctions near the basement membrane of the seminiferous epithelium (14). The local environment of the testis is immunosuppressive and is characterized by the expression of TGFβ1-β3, IL-10, and activin A, the last of which inhibits the expression of IL-1 and IL-6 (13). Additionally, androgens are believed to play a role in maintaining the local immunosuppressive environment within the testis (13). Testicular macrophages and dendritic cells are less responsive to antigens than they are in other tissues (15, 18). Furthermore, the testis contains regulatory T cells, which attenuate the immune response (19).

## Cancer Privilege

More recently, cancers have been identified as immune privileged sites. Cancer cells contain mutations that produce antigens which appear foreign to the host immune system. Consequently, immune privilege is advantageous in cancer because it allows the cancer to evade the host immune system (20). There are a very large number of possible cancer mutations, and each type of cancer may possess unique mechanisms of immune privilege depending on the mutations it has accrued (21). Generally, cancers can avoid the host immune system by downregulating the activation of T cells through the expression of checkpoint proteins, expressing enzymes and ligands that induce the apoptosis of T cells, and maintaining an immunosuppressive microenvironment (21, 22). Cancers may express checkpoint molecules such as cytotoxic T-lymphocyte-associated protein 4 (CTLA-4) and programmed cell death protein ligand 1 (PD-L1), which negatively regulates the activation of T cells (21, 22). Cancers can also express other proteins such as FasL and IDO, which can induce T cell apoptosis (21, 22). Additionally, the immunosuppressive microenvironment of cancers can be characterized by the upregulation of anti-inflammatory cytokines, such as IL-10 and TGFβ (21, 22). These cytokines limit the activation of the host immune response.

## Immune Privilege of Heart Valves

**Figure 2:**
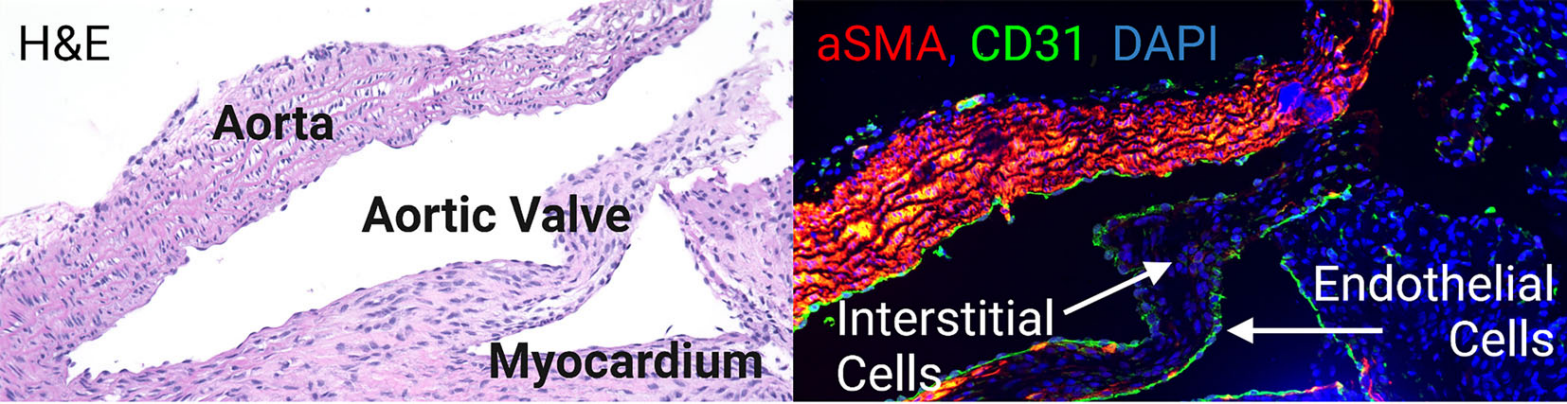
The aortic valve is in continuity with the aorta and the myocardium. It is composed of an endothelial lining and an interstitial core.

Heart valves share many features with the immune privileged sites discussed above. Like classic immune privilege sites, the heart valves are of vital importance to the organism, ensuring that blood flows in a single direction from the heart, through the lungs, and to the rest of the body. Furthermore, heart valves, which are composed of an interstitial core lined by endothelium, have limited regenerative capacity (**Figure 2**). Injury to these tissues has potentially immediate fatal cardiac or systemic consequences (23). Therefore, similar to immune privileged sites, very little inflammation can be tolerated in the heart valves without detrimental effects for the organism. In fact, because heart valves cannot regenerate spontaneously, diseases involving the heart valves frequently necessitate surgical replacement (23). The earliest surgical implants used for valve replacement were homografts. Aortic homografts have been inserted into the subcoronary position since 1962 (24, 25). Contrary to organ transplants which require immediate and lifelong immunosuppression, allograft heart valve implants display long-term functionality without HLA and ABO matching and without immunosuppression of the recipient (26). Furthermore, even transplanted hearts that have failed due to rejection have been shown to have functionally and structurally intact semilunar valves, suggesting valves may be immunologically distinct from heart tissue (27–29). Several other studies have noted the aortic valve as a possible immune privileged site (30–32). In a study done by Mitchell and colleagues looking at the aortic valves from transplanted hearts, they found the aortic valves maintained near-normal overall architecture and cellularity, without apparent immunologic injury, even in the setting of fatal myocardial rejection or graft arteriosclerosis (27). When they looked at explanted cryopreserved allografts in the same study, they found no differences in valves from allografts failing due to severe parenchymal rejection regardless of immunosuppression regimen, nor in valves from allografts having as many as 12 rejection episodes. They therefore concluded that there was no valvular destruction, dysfunction, or morphological effect resulting from cell-mediated immune injury (27).

A similar study by Valente and colleagues also looked at the aortic valve after heart transplantation and found that valve competence and cusp pliability were maintained even in long-term specimens, with no cases of valve dysfunction or significant calcification (28). Interestingly, the study noted concomitant subendothelial lymphocytic infiltrates and aortic valve edema in cases with acute myocardial rejection, however this did not appear to compromise the long-term viability or durability of the valve. Tissue viability was confirmed by histology and showed perfectly preserved fibroblasts both early and in the long-term (28).

Similarly, O’Brien and colleagues were able to demonstrate long-term viability of homografts with chromosomal analysis that demonstrated the persistence of male donor cells in culture of fibroblasts from a valve leaflet removed over 9 years after implantation in a female recipient, indicating donor cells are able to survive and proliferate without destruction by the host immune system (33). Another study performed by O’Brien et al. examining aortic valves from six transplanted hearts found that while there was some evidence of interfibrillary edema and mild cellular infiltrates, the general appearance was essentially that of normal, viable valves (29).

Furthermore, Balch et al. concluded that blood group rhesus mismatch are not associated with an increased risk of valve degeneration in fresh allograft valves (34).

Several animal studies have also indicated that aortic valves may be immunologically distinct from other cardiac tissues. Chen et al. demonstrated that while porcine hearts transplanted into baboon recipients were hyperacutely rejected within hours after implantation, the aortic and pulmonary valves were entirely spared, with no signs of IgM- or membrane attack complex- mediated damage (32). Another study reported no microscopic evidence of rejection in fresh canine aortic valve allografts transplanted to subcoronary sites when the animals were pre-sensitized with skin grafts after 10 days (30). Additionally, Heslop et al. found no acceleration of skin graft rejection in rats who received subcutaneous implantation of aortic valve leaflets (35). In attempt to delineate the role of immune-mediated injury in allograft valve failure, several animal models were utilized and found that aortic valves heterotopically implanted into the abdominal aorta resulted in greater inflammatory infiltration and structural deformity in allogeneic transplants as compared to syngeneic ones (31, 36, 37). Other studies using similar models showed daily treatment with cyclosporine prevented leaflet structural integrity and inhibited cellular infiltration, suggesting that early valve leaflet failure is immune mediated (38, 39). However, of note, to prevent local thrombosis in these animals models, one valvular cusp is often rendered incompetent to ensure the sinuses of Valsalva are washed out (40, 41). Therefore, since the valves are non-functional, they are not satisfactory models to study the effect of alloreactivity on the function of aortic valve allografts. Furthermore, these heterotopically placed aortic valves typically fail by retrovavular thrombosis, which is used as a maker for immune-mediated damage in these studies. However, thrombosis in human allografts is rare, indicating their findings may not be clinically translatable. Additionally, to implant the aortic valve into the abdominal aorta, a relatively greater length of aorta as well as the attached myocardium on the ventricular side of the valve graft must be retained, which are important sources of antigen that may be contributing to the immune response detected in these models (31).

Others have proposed that heart valves are in fact antigenic and capable of eliciting an immune response in its host (42–44). Moreover, while the fact that homografts are usually transplanted without matching the donor and recipient for blood group or human leukocyte antigens and without immunosuppression points to a potential immune privilege-like property of semilunar valves, homografts are prone to failure, particularly in children. In children younger than 3, allograft failure may be as high as 70% with a mean replacement interval of 1.9 years after the original operation (45). Interestingly, in one study looking at failed homografts, all the valves examined from infants contained a lymphocytic infiltrate, while the failed homografts from adults showed leaflet calcification and fibrosis but no inflammation (46). While several factors such as preparation, handling, hemodynamic, technical, or infectious causes may contribute to this deterioration, many suggest an immunologic basis for failure of aortic valve allografts despite previous notions that valves are spared from rejection. For instance, cryopreserved allografts have been found to induce a detectable donor HLA-specific humoral response, however the location of induction and amplification of the immune response to aortic valve allografts remains less certain (47).

Most regard valvular endothelial cells as the main target cells in the immune response directed against aortic valve allografts (42, 44, 48). Li et al. demonstrated an immune response to homograft valve endothelial cells both *in vivo* and *in vitro*, and found that FasL can be overexpressed in endothelial cells to modify the cells’ immunological behavior (48). Another study found valvular endothelial cells expression of intercellular adhesion molecules-1 (ICAM-1) and E-selectin, which play critical roles in peripheral blood mononuclear cell attachment and initiation of immune responses (44). Interestingly, the same study found cryopreserved allograft valves were almost always completely denuded while valves obtained from previous transplanted hearts contained a confluent layer of valvular endothelium with no signs of re-endothelization by the recipient, indicating they were resistant to degeneration when implanted fully viable, without previous cryopreservation, into a recipient receiving systemic immunosuppresion (44). However, despite evidence that the endothelial cells act as an antigenic surface for the valves and may contribute to long-term degeneration, the use of valves containing viable endothelial cells have resulted in significant improvements in the long-term performance of homografts (49).

Therefore, one the one hand, aortic valve allografts have successfully been implanted donor-recipient matching or immunosuppression, and aortic valves from transplanted hearts appear to be relatively spared from immune-mediated damage, indicating valves may have immune-privilege. It is therefore reasonable to conclude there is something immunologically distinct about the valve itself or the sub-coronary position.

On the other hand, there is a body of evidence that aortic valves are in fact immunogenic and capable of eliciting a donor-specific immune response in the recipient. However, it is unknown whether or to what extent this alloreactivity ultimately results in valve allograft failure.

## Possible Mechanisms for Heart Valve Immune Privilege

There are several potential mechanisms that may contribute to heart valves’ relative resistance to immune injury. These properties may be a result of intrinsic characteristics of the valve itself or as a result of anatomical and physiologic aspects of the sub-coronary position.

Some have postulated that the homologous aortic valve may be inherently un-immunogenic or demonstrate low antigenicity (30, 35). Interestingly, Heslop et al. found that whole aortic valve allografts implanted into the abdominal aorta unequivocally mounted an immune response while valve leaflets alone were ineffectual (35). They concluded antigenicity resides predominantly in the rim of the cardiac muscle, while allogenic aorta elicited a less pronounced response and valve leaflets were not demonstratable immunogenic. They also showed the antigenicity of whole aortic valve grafts was low in comparison to other tissues such as skin, kidney, and heart muscle (35). Mousthapha and colleagues also commented on the importance of the transplanted myocardial cuff, stating that the massive infiltration of polymorphonuclear leukocytes seen in both syngeneic and allogeneic grafts may be a result of a non-specific inflammatory reaction caused by ischemic necrosis of the myocardial cuff. This PMN spill over resulting in medial cell loss may be the source of the release of cytokines and growth factors resulting in the up-regulation of cell adhesion molecules, which may contribute to the initial infiltration of lymphocytes or potentiate thrombosis (36). Mitchell et al. similarly postulated that the lower metabolic demand of valves compared to myocardium may result in less ischemic injury and consequently less ischemia-induced up-regulation of adhesion molecules (27).

While aortic valve leaflets themselves may be less antigenic than their surrounding tissue, finite quantities of myocardium and aorta are necessarily implanted with the valve in human valve transplantation (31). Additionally, with the mounting evidence that aortic allograft valves are in fact antigenic, another explanation must be considered to explain valves’ seemingly resistance to rejection (31, 42, 44, 48, 50–52). It is reasonable to theorize that valve allografts may be a low-visibility target for the host immune system because no microvasculature develops between the host and the graft (31, 46, 50). In addition to the lack of blood vessels in cardiac valves, the hemodynamics of the sub-coronary position may also have a protective factor. The aortic valve is constantly in motion within a fast-moving stream, with washing jets from the sinuses of Valsalva. This high-pressure and high flow over the valves, relative to slower flow within the myocardium, may nullify the chemotactic response (27, 53). Additionally, protein expression is closely regulated in endothelial cells and may be modulated by the shear force exerted on valvular endothelium. Specifically, expression of proteins such as major histocompatibility complex antigen and vascular cell adhesion molecule 1 are known to be influenced by flow rate (54, 55). Interestingly, in a study done in canines, subcutaneously implanted homologous aortic valves showed infiltration by plasma cells and lymphocytes while no round cell infiltration characteristic of immune reaction was demonstrated in orthotopically transplanted leaflets, suggesting something unique about the subcoronary position—perhaps the hemodynamic of the environment (30).

Furthermore, it is possible that leaflet tissue has a distinct mechanism of trafficking effector cells. One study found that treatment with anti-a4/b2 integrin resulted in significant reduction in leaflet infiltration by macrophages, T-cells, and CD8+ T cells in a rat model (38). However, the same blockade was unable to reduce monocyte or macrophage infiltration in the adventitia of the graft, indicating valve tissue may have a distinct pathway for immune cell chemotaxis that differs from its surrounding tissue.

Yet another explanation for the relative immune sparing properties of heart valves may lay within the valve interstitial cells. Valve endothelial cells and interstitial cells express similar levels of human leukocyte antigens and adhesion and costimulatory molecules, however, it is only the endothelial cells that are immunogenic (50). In fact, while T-cell responses to endothelial cells were detected after interferon gamma treatment, no response was detected after interferon gamma-treated interstitial cells, resulting in the induction of T-cell anergy (50). Moreover, the addition of the costimulatory molecule B7-1 restored full T-cell activation against the valve interstitial cells, suggesting these cells are deficient in a costimulatory factor which appears to be present on endothelial cells. It is therefore possible that the donor-specific T-cell anergy induced by valve interstitial cells may counteract the immunogenic valvular endothelial cells, resulting in the relative immune sparing phenotype observed in heart valves.

**Figure 3:**
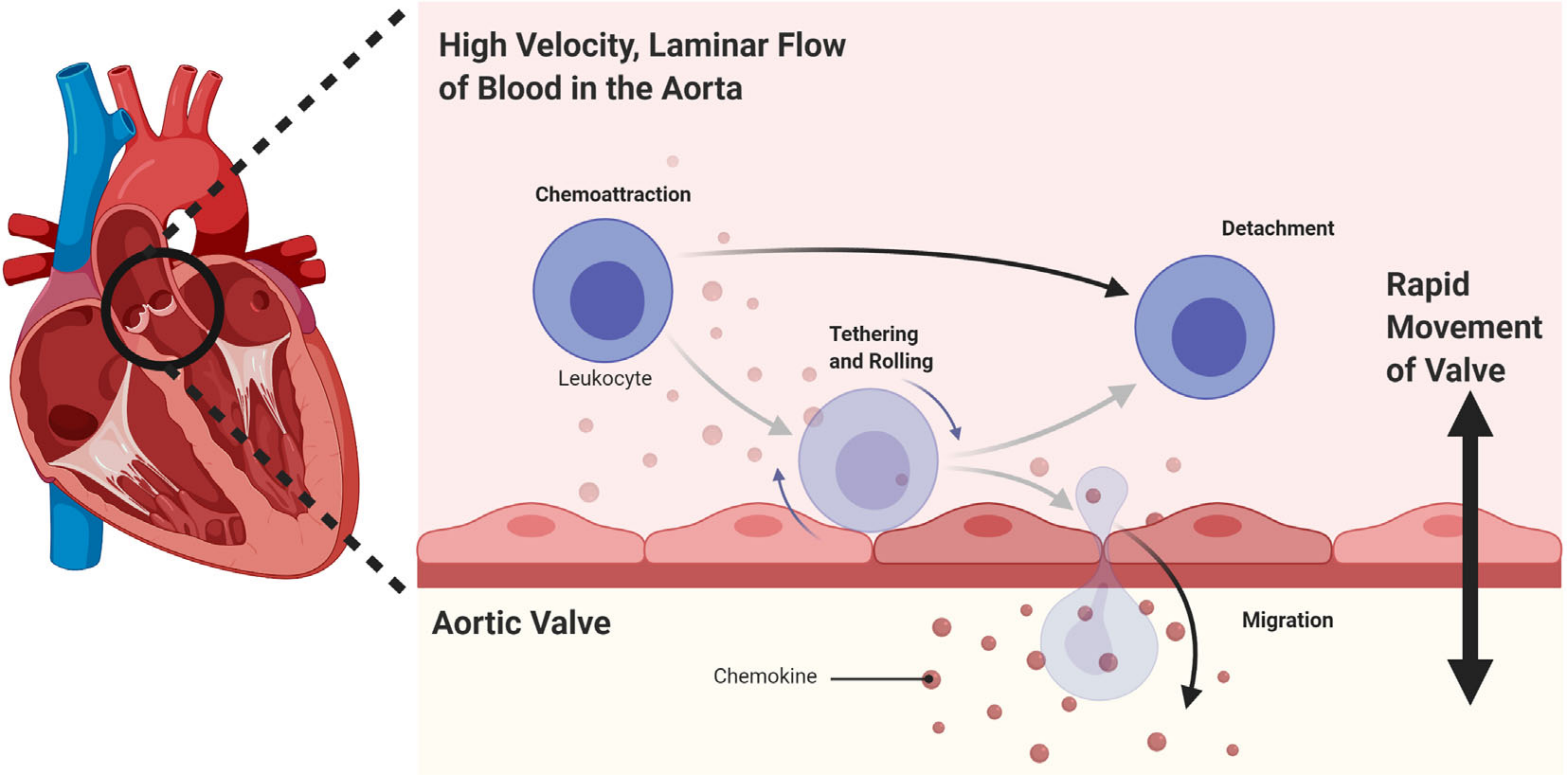
A possible mechanism for the immune privilege of heart valves could be inhibition of immune cell extravasation in the context of rapid blood flow in the aorta, combined with the vigorous movement of the opening and closing valve leaflets.

Finally, a possible unique mechanism for the immune privilege of heart valves could be inhibition of immune cell extravasation in the context of rapid blood flow in the aorta, combined with the vigorous movement of the opening and closing valve leaflets (**Figure 3**).

## Clinical Implications of Immune Privilege

Immune privilege has important clinical implications. With immune privileged sites, there are fewer immunogenic processes that need to be disrupted to prevent rejection. This is exemplified by corneal transplantation. Because there is little to no rejection seen in corneal transplantation, it has become the most successful human solid organ transplantation (56). In the cornea, hemangiogenesis and lymphangiogenesis, immune-modulating factors and the immunogenic potential of the corneal tissue can all be inhibited using local agents without the need for systemic immune suppression. Topical antagonistic antibodies, localized gene therapy and inhibitory immune checkpoint expression can all be used (2). These types of immunosuppression reduce the need for systemic immunosuppression because they can be applied to the corneal tissue only (57). Once the donor cornea is implanted in the recipient, the anatomic, molecular and cellular barriers of the eye, such as the blood-ocular barrier, prevent a majority of immune molecules and cells from accessing the site, reducing the number of immunogenic processes that occur (56).

Knee meniscal transplantation is similar to corneal transplantation because the meniscus is another immune privileged site in the body. There are no adverse immunological reactions or allograft transplant rejections seen in animal models (58, 59). This transplantation has been shown to decrease pain, reduce swelling and be chondroprotective by improving peak contact stresses and total contact area in the knee in humans (60, 61). Both corneal and knee meniscal transplantation greatly improve the quality of life of patients through restoring vision and ambulation, respectively, in addition to reducing pain. The potential clinical implications of transplanting immune privileged heart valves are huge because they could save lives of patients with disease valves, improve the quality of life they lead and decrease the need for systemic immunosuppression and decrease the associated side effects.

Immune privilege of heart valves also has important clinical implications. For example, allograft heart valve implants display long-term functionality without HLA and ABO matching even in the absence of immunosuppression of the recipient (26). Moreover, it raises the possibility that heart valve transplants may grow adaptively with children with limited or no need for immune suppression (62).

## Conclusion

Immune privilege is a spectrum of evolutionary adaptations that protect vital tissues with limited regenerative capacity from collateral damage by the immune response. There is evidence that heart valves are on the spectrum of immune privileged sites. The mechanism could be related to inhibition of immune cell extravasation in the context of rapid blood flow combined with the vigorous movement of the opening and closing leaflets. This also has important clinical implications for heart valve transplantation.

## Author Contributions 

All authors listed have made a substantial, direct, and intellectual contribution to the work, and approved it for publication.

## Funding

This work was supported in part by the AATS Foundation Surgical Investigator Grant to TKR, the Children’s Excellence Fund held by the Department of Pediatrics at the Medical University of South Carolina, the Emerson Rose Heart Foundation, NIH-NHLBI Institutional Postdoctoral Training Grants (T32 HL-007260), and the FLEX Funds for Research/Scholarly Projects through the Medical University of South Carolina College of Medicine. Figures were created with BioRender.com.

## Conflict of Interest

The authors declare that the research was conducted in the absence of any commercial or financial relationships that could be construed as a potential conflict of interest.

## Publisher’s Note

All claims expressed in this article are solely those of the authors and do not necessarily represent those of their affiliated organizations, or those of the publisher, the editors and the reviewers. Any product that may be evaluated in this article, or claim that may be made by its manufacturer, is not guaranteed or endorsed by the publisher.
